# HE4 overexpression decreases pancreatic cancer Capan-1 cell sensitivity to paclitaxel via cell cycle regulation

**DOI:** 10.1186/s12935-020-01248-1

**Published:** 2020-05-12

**Authors:** Fengbiao Guo, Jinping Li, Yaozhi Qi, Jianqing Hou, Haibin Chen, Shi-Wen Jiang

**Affiliations:** 1 Department of Histology and Embryology, Shantou University Medical College, Shantou, 515041 Guangdong China; 2 Center of Reproductive Medicine, The Affiliated Wuxi Maternity and Child Health Care Hospital of Nanjing Medical University, Wuxi, 214123 Jiangsu China; 3 Department of Biomedical Science, Mercer University School of Medicine, Savannah, GA 31404 USA; 4 Department of Surgery, Anderson Cancer Center, Memorial Hospital University Medical Center, Savannah, GA 31404 USA; 5 Department of Clinical Laboratory, Lianyungang Maternal and Child Health Hospital, Lianyungang, 222005 Jiangsu China; 6 Department of Obstetrics and Gynecology, Qingdao University Medical College Affiliated Yantai Yuhuangding Hospital, Yantai, 264000 Shandong China

**Keywords:** HE4, Paclitaxel, Pancreatic cancer, Cell cycle, Drug sensitivity

## Abstract

**Background:**

Paclitaxel is a first-line chemotherapy drug for pancreatic, ovarian, endometrial cancers and other malignancies. However, its efficacy is often compromised by decreased cell sensitivity or the development of resistance. Human epididymis protein 4 (HE4) is highly expressed in gynecologic and pancreatic cancer tissues, and its serum levels are used for patient triage and assistant diagnosis of gynecologic cancers. Previous studies have shown that HE4 overexpression could promote cancer cell proliferation and the growth of tumor xenografts, which suggests its potential involvement in cancer chemosensitivity.

**Methods:**

Two pancreatic cancer cell lines, Capan-1 and Suit-2, were transiently transfected with an HE4 overexpression plasmid, and transfected cells were treated with paclitaxel. S-phase cells were labeled using BrdU, and cell positivity rates were determined by counting BrdU-positive cells. Following HE4 overexpression and/or drug treatment, a western blotting analysis was performed to determine the protein alterations of PCNA and p21, two important cell cycle regulators.

**Results:**

HE4 overexpression not only promoted the proliferation of the Capan-1 pancreatic cells, but also significantly decreased cell sensitivity to paclitaxel. Results from western blotting showed that paclitaxel inhibited cell proliferation by decreasing the expression of PCNA and increasing the expression of p21. Data analysis indicated interactive actions between HE4 function and paclitaxel effects, both converging to cell cycle regulation.

**Conclusion:**

These findings suggest that HE4 could be a potential therapeutic target for the sensitization of pancreatic cancer cells to paclitaxel treatment. HE4 expression levels may be used to predict the sensitivity of pancreatic cancer patients to paclitaxel.

## Background

Pancreatic cancer is one of the most aggressive and lethal malignancy. While significant improvements have been made in the management of other cancers, the mortality of pancreatic ductal adenocarcinoma (PDAC) remains high, with a 5-year survival rate of 7% [1, 2], and ranks as the seventh leading cause of cancer deaths globally [3]. The chemotherapy regimen for PDAC is not effective in many cases due to the poor response or development of drug resistance [4, 5]. Currently, the chemotherapy for PDAC consists of the combined use of paclitaxel (Pac) or 5-fluorouracil with gemcitabine [6, 7]. Novel therapies such as enzalutamide alone or in combination with gemcitabine and albumin-bound paclitaxel particles (nab-paclitaxel) were shown to be effective in murine models of PDAC [8, 9]. A Phase I/II clinical trial performed by Sahai et al. [10] has shown preliminary efficacy of metronomic 5-fluorouracil plus nab-paclitaxel in metastatic pancreatic cancers. However, drug resistance continues to be a major impediment for PDAC treatment [11]. There is an urgent need to explore a novel strategy to overcome the chemoresistance of pancreatic cancers.

Generally, several potential mechanisms dictate drug sensitivity or the development of resistance to chemotherapy: (1) The intracellular concentrations of small, hydrophobic, nonpolar drug molecules can be reduced by cell membrane pumps through efflux mechanisms. It is well recognized that the overexpression of the P-glycoprotein (MDR1) leads to increased drug resistance [12, 13]. (2) Drug resistance is associated with modifications of drug metabolism, e.g., changes in the conversion of cytotoxic nucleoside and nucleotide derivatives by kinase and phosphoribosyl transferase [14]. (3) Increased DNA repair could promote cell tolerance to DNA damage drugs [15]. (4) Expression alterations or post-translation modifications of key cell cycle or apoptosis regulators could lead to changes in cell sensitivity to drugs targeting these pathways. A wide range of factors participate in cell cycle/apoptosis modulation, and identification of such factors is required for a better understanding of chemoresistance mechanisms and the improvement of PDAC treatment.

Human epididymis protein 4 (HE4) is a secretory glycoprotein belonging to the whey-acidic-protein (WAP) family containing the WAP-type four-disulfide core (WFDC) domains [16]. HE4 protein is constitutively expressed in the human epididymis, trachea, ovary, and endometrium [17–20]. HE4 overexpression was detected in ovarian and endometrial cancers as well as a variety of non-gynecologic cancers [21, 22]. An HE4 serum test has been used as a biomarker for the triage of patients suspected for gynecologic cancers, and for assistant diagnosis and prognosis of ovarian and endometrial cancers. Moore et al. [23] conducted a follow-up study with 89 ovarian cancer patients and showed that patients with serum HE4 levels < 500 pM had a 5-year survival rate of 59%, but patients with HE4 levels > 500 pM had a poor 5-year survival rate of 27%. Huang et al. [24] found that human pancreatic carcinoma tissues expressed higher HE4 levels than normal pancreatic tissues or adjacent non-tumor tissues. HE4 levels were inversely correlated with the clinical stages of PDAC. Moreover, the combined use of HE4 with CA19-9 and CA15-3 could improve the diagnosis of PDAC. Indeed, several in vitro and in vivo studies have shown that HE4 possesses bioactivities in cancer cells. Li et al. [25] reported that HE4 overexpression in Ark2 endometrial cancer cells enhanced several malignant phenotypes, including increased cell proliferation, Matrigel invasion, and colony formation in soft agar. Besides, HE4 overexpression promoted tumor growth in a mouse xenograft model. Moore et al. [23] compared mouse xenograft tumor sizes formed by HE4-overexpressing ovarian cancer SKOV-3 cells and cells without HE4-overexpression, and found that the former grew significantly larger tumors than the latter. These findings strongly suggested that HE4 may play an active role(s) in the regulation of cancer cell behavior.

Our preliminary studies on multiple chemotherapy drugs indicated that cell sensitivity to paclitaxel was related to HE4 expression levels in pancreatic cancer cell lines. Paclitaxel is widely used in the treatment of pancreatic, breast, non-small cell lung cancers and gynecologic cancers [26]. Paclitaxel stabilizes microtubule and protects it from disassembly in cell division, resulting in cell cycle arrest. Recently, Lu et al. [27] found that treatment of pancreatic and endometrial cancer cell lines with a relatively low concentration of purified, extracellular HE4 protein led to increased cell proliferation and DNA synthesis as well as alterations in cell cycle progression. These observations point to a possibility that HE4 may have an impact on paclitaxel resistance via its action in cell cycle regulation. Investigation along this line will help us to better understand HE4 interaction with drug sensitivity, which may facilitate the development of new treatment modalities to overcome the resistance of PDAC to paclitaxel treatment.

## Materials and methods

### Immunohistochemistry

The PDAC tissue microarray array was purchased from US Biomax (Rockville, MD, USA). Array slides were deparaffinized with 3 changes of Xylene, each for 5 min. The slides were rehydrated with a sequential treatment of 100% ethanol and 95% ethanol, each for 2 changes and 5 min, which was followed by one change of 80% and 75% ethanol, each for 5 min. Endogenous peroxidase activity was quenched with 3% H_2_O_2_ at room temperature for 30 min. After rinsing with distilled water, the slides were incubated at 98 °C for 30 min in Epitope Retrieval Buffer (IHC-101, Bethyl Laboratories, Montgomery, TX, USA). After incubation in blocking solution for 30 min, slides were exposed to anti-HE4 rabbit polyclonal antibody (1:350, Cat. No. Ab109298, Abcam Cambridge, MA, USA) for 1 h at room temperature. The anti-rabbit secondary antibody from the above kit was applied at 1:400 dilutions for 1 h. Color development was performed with DAB Substrate (IHC-101, Bethyl Laboratories, Inc., Montgomery, TX, USA). Counterstaining was carried out with Gill’s Hematoxylin Solution (Cat. No. SC-24973, Santa Cruz Biotechnology, Dallas, TX, USA).

### Cell culture and cell counting

Pancreatic cancer cell lines Capan-1 and Suit-2 were obtained from the American Type Culture Collection (ATCC). Cells were maintained in RPMI-1640 medium (HyClone Laboratories, Inc., Logan, UT, USA) containing 10% fetal bovine serum (Cat. No. 10100154, Thermo Scientific, Waltham, MA, USA) and 1% penicillin/streptomycin (100X; Mediatech, Inc., Manassas, VA, USA), at 37 °C and a 5% CO_2_ atmosphere. Paclitaxel was purchased from Sigma-Aldrich Co. (Merck KGaA, Darmstadt, Germany).

Capan-1and Suit-2 cells were cultured in 48-well plates. One day after transient transfection, the cells were treated with paclitaxel (20 nM, final concentration) for 96 h, and cell numbers were counted every 24 h. At each time point, cells were collected by digestion with 1× trypsin–EDTA. Suspended cells were stained with 0.4% trypan blue solution, and the cell number was counted on the hemocytometer under a microscope. The assay was performed in triplicates, and data were presented as mean ± SD.

### MTT assay

The MTT method was used to measure cell viability. Capan-1 cells transfected with the empty vector were divided into two groups, one treated with 20 nM Pac (Pac group), and another treated with DMSO solvent as control (Ctrl). Similarly, cells transfected by HE4-expressing plasmid DNA were also treated with DMSO solvent (HE4) or 20 nM of Pac (HE4 + Pac). Following cell transfection and Pac treatment, 20 μl of CellTiter 96 A Queous One Solution Reagent (Cat. No. G5430, Promega, Madison, WI, USA) was added to the culture medium for MTT assay. One hour later, 100 μl culture medium was removed from the plate, and the absorbance at 490 nm was measured on a 96-well plate reader (Modulus Microplate Multimode Reader, Turner BioSystems, USA). Each experiment was performed in triplicates, and the final results were presented as mean ± SD.

### BrdU incorporation assay

Cells were seeded on two-chamber slides (1 × 10^5^ cells/well) and cultured under standard conditions. Following transfection and treatment with paclitaxel (20 nM, final concentration) for 48 h, 5-bromo-2′-deoxyuridine (BrdU) (Cat. No. 000103; Invitrogen, Rockville, MD, USA) was added into the cell culture to label the S-phase cells for 2 h. Cells were washed with cold phosphate-buffered saline (PBS), fixed in 70% alcohol for 30 min at 4 °C, and permeabilized with 0.2% Triton X-100 for 30 min. Immunocytochemistry was performed using the BrdU staining kit (Cat. No. 933943; Invitrogen) following the manufacturer’s instructions. Images were captured with the Nikon DS-Fi2 camera (Nikon Instruments, Inc., Melville, NY, USA). The BrdU-positive cells were counted and BrdU incorporation rates were calculated. The assay was repeated three times and final results were presented as mean ± SD.

### Western blotting analysis

Cell culture was washed twice with cold PBS (pH 7.4) and lysed on ice with cold lysis buffer (150 mM NaCl, 50 mM Tris–HCl (pH 7.4), 0.1% SDS, 0.5% sodium deoxycholate, 1% Nonidet P-40) supplemented with 1% Halt protease inhibitor cocktail (100X; Thermo Scientific, Rockford, IL, USA), 1 mM phenylmethylsulfonyl fluoride (PMSF), 5 mM sodium fluoride (NaF), and 1 mM sodium vanadate (Na_3_VO_4_). Cell suspensions were kept on ice for 30 min before centrifugation at 12,000 rpm at 4 °C. The supernatants were collected and stored at − 80 °C until use. Forty micrograms of protein were mixed with SDS-loading buffer, boiled for 5 min, separated in 12% SDS–polyacrylamide gels, and transferred onto PVDF membranes. The non-specific reaction was blocked for 1 h at room temperature in TBST containing 5% non-fat milk and 0.1% Tween-20. Primary antibodies (anti-HE4 rabbit polyclonal antibody, 1:3000, Cat. No. Ab109298, Abcam Cambridge, MA, USA; anti-PCNA mouse monoclonal antibody, 1:1000, Cat. No. SC-56, anti-p21 mouse monoclonal antibody, 1:1000, Cat. No. SC-397, Santa Cruz Biotechnology, Inc. Dallas, Texas, USA) diluted in blocking solution were applied and incubation was carried out at room temperature for 1 h. Secondary antibodies (goat anti-rabbit IgG-HRP, Cat. No. SC-2301, Santa Cruz Biotechnology, Inc. Dallas, Texas, USA; goat anti-mouse IgG-HRP, Cat. No. G21040, Thermo Scientific, Waltham, MA, USA) were 1:10,000 diluted and incubation was performed at room temperature for 45 min. The blots were developed with the ECL system (Cat. No. 32106, Thermo Scientific, Rockford, IL, USA) and exposed to X-ray film. Membranes were stripped and GAPDH was detected with anti-GAPDH rabbit monoclonal antibody (1:1000, Cat. No. 70R-12079, Fitzgerald Industries International, Acton, MA, USA). Results from GAPDH detection served as protein loading controls.

### Manipulation of HE4 expression level

**Fig. 1:**
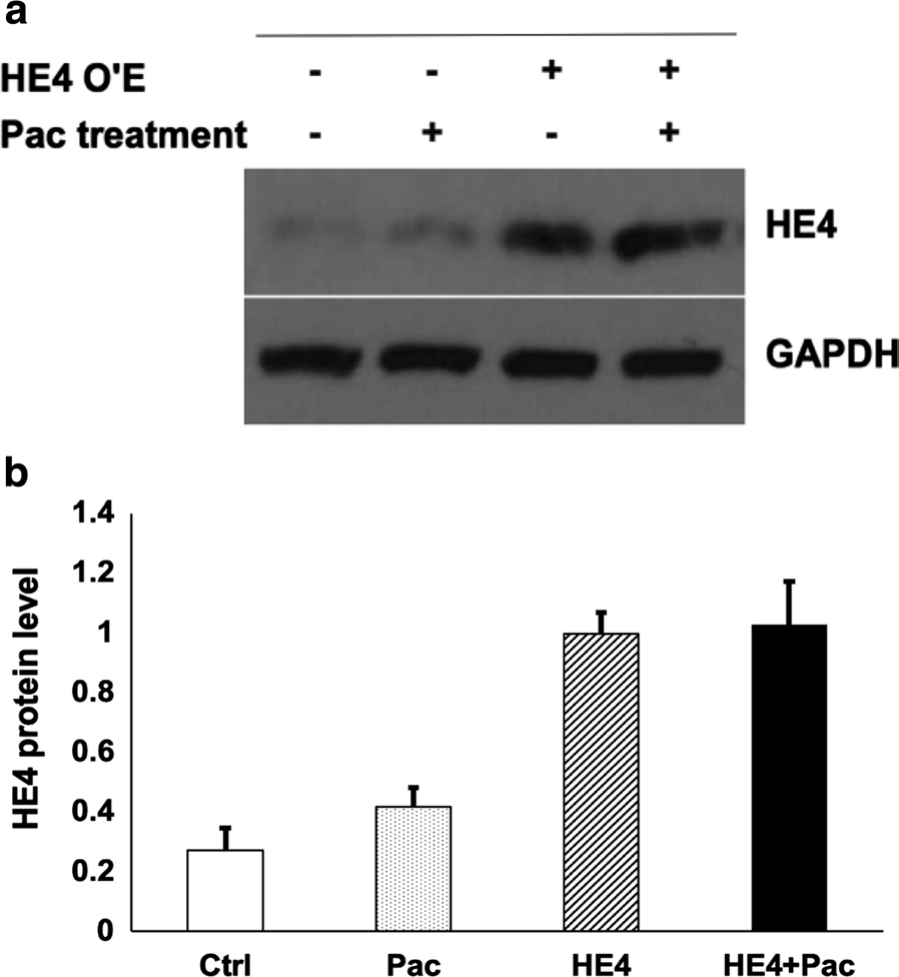
HE4 protein expression levels in the four experimental groups of control (Ctrl), HE4 overexpression (HE4), Pac treatment (Pac), and overexpression plus Pac treatment (HE4 + Pac). Western blotting was performed with HE4- and GAPDH-specific antibodies, respectively, following cell transfection with HE4 expression plasmid DNA and/or Pac treatment. **a** Representative results of the Western blotting analysis for HE4 and GAPDH. **b** The results were scanned for densitometry analysis. Levels of HE4 were normalized with those of GAPDH, the protein loading marker. A more than three-fold increase in HE4 level was achieved by the overexpression manipulation. Note that HE4 levels remained stable following Pac treatment

**Fig. 2:**
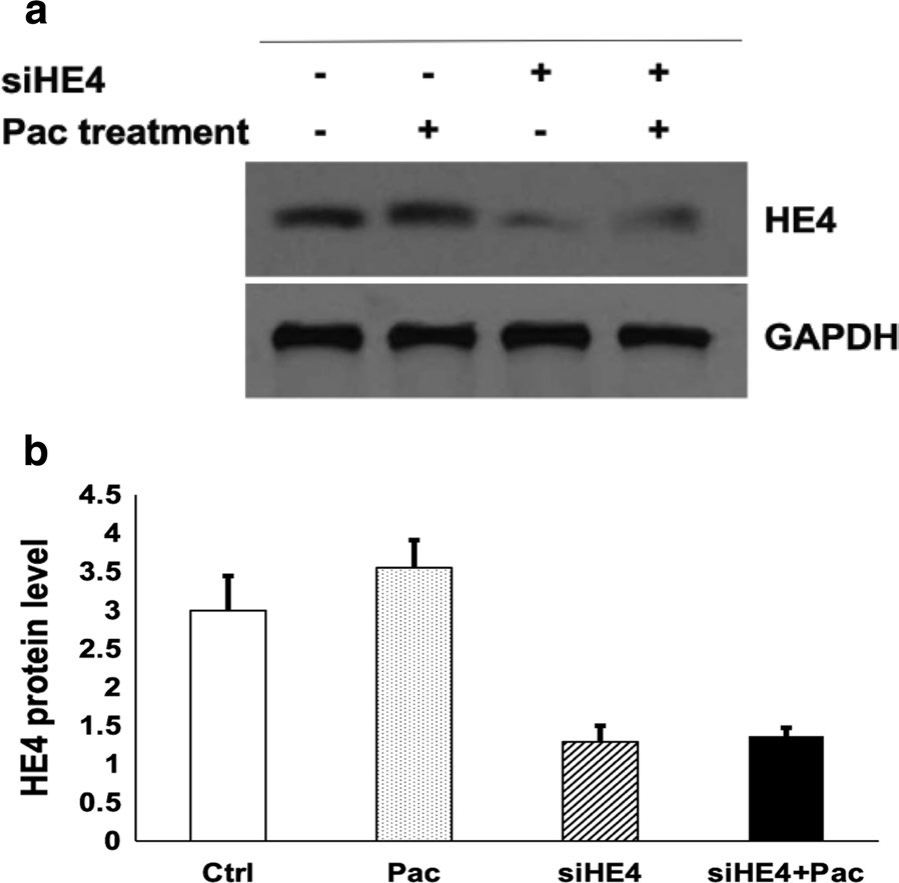
HE4 protein expression levels in control (Ctrl), HE4 knockdown with siRNA (siHE4), Pac treatment (Pac), and knockdown plus Pac treatment (siHE4 + Pac) groups. Western blotting was performed with HE4- and GAPDH-specific antibodies, respectively, following cell transfection with HE4 expression plasmid DNA or Pac treatment. **a** Representative results of Western blot analysis for HE4 and GAPDH. **b** The results were scanned for densitometry analysis. Levels of HE4 were normalized with those of GAPDH. A nearly 60% reduction in HE4 level was achieved by siRNA-mediated knockdown. HE4 levels remained stable following Pac treatment

The pcDNA 6.0 plasmid was used to construct a vector for HE4 overexpression. For HE4 knockdown, two HE4-specific small interfering RNA (siRNA) species were designed by IDT (Integrated DNA Technologies, Inc. Skokie, IL, USA), synthesized (Cat. No. 301750, QIAGEN, Valencia, CA, USA), and tested by direct transfection into cultured cells. Real-time PCR was performed to verify the changes in HE4 expression. The efficacy of HE4 expression manipulation was confirmed with the use of Western blotting. As shown in Fig. 1, approximately a three-fold increase in HE4 protein levels was achieved by transient transfection with a plasmid overexpressing HE4. A more than 60% reduction of HE4 protein was observed when cells were transfected with HE4-siRNA (Fig. 2). Moreover, Pac treatment did not appear to significantly affect the HE4 expression levels in either overexpression or knockdown experiments.

### Statistical analysis

For quantitative data, means and standard deviations were calculated for each experimental group. Student’s t-tests were performed using SPSS 17.0 software (SPSS, Inc., Chicago, IL, USA) and p ≤ 0.05 was considered a level of statistical significance.

## Results

Since the current study dealt with two factors from different angles—a chemotherapy drug, and a cellular factor HE4—to delineate their individual effects and the potential interaction, all the experiments were designed to include four groups: (1) Control without Pac treatment and HE4 expression manipulation (labeled as Ctrl in figures). The experimental read-out from this group, e.g., a BrdU incorporation rate, can be represented by value “A”; (2) Pac treatment alone (labeled as Pac in figures). The experimental read-out from this group is represented by value “B”; (3) HE4 manipulation alone, overexpression (labeled as HE4 in figures) or knockdown (labeled as siHE4 in figures). The experimental read-out from this group is represented by value “C”; (4) HE4 expression manipulation plus Pac treatment (labeled as HE4 + Pac or siHE4 + Pac in figures). The experimental read-out from this group is represented by value “D”.

The quotient of B/A will indicate the Pac drug effect alone in the absence of HE4 manipulation. Similarly, C/A represents the effect of HE4 expression manipulation alone in the absence of drug treatment. D/C represents the Pac effect in the presence of HE4 manipulation. Importantly, we can calculate the value “X”: X = (D/C)/(B/A). This value will tell if the two factors act independently or interactively. An X value around 1 would indicate independent actions in a non-interactive manner by the two factors, no matter how much D/C or B/A changes by themselves, or in other words, HE4 manipulation does not affect the drug sensitivity. In contrast, an X value deviating from 1would indicate interaction by the two factors. For those negative effects by drug treatment, e.g., the Pac-caused inhibition of cell proliferation rate, an X value higher than 1 will indicate that HE4 manipulation could counteract the drug effects, while an X value lower than 1 will suggest synergism between HE4 manipulation and drug treatment.

### Pac and HE4 effects on cell proliferation

**Fig. 3:**
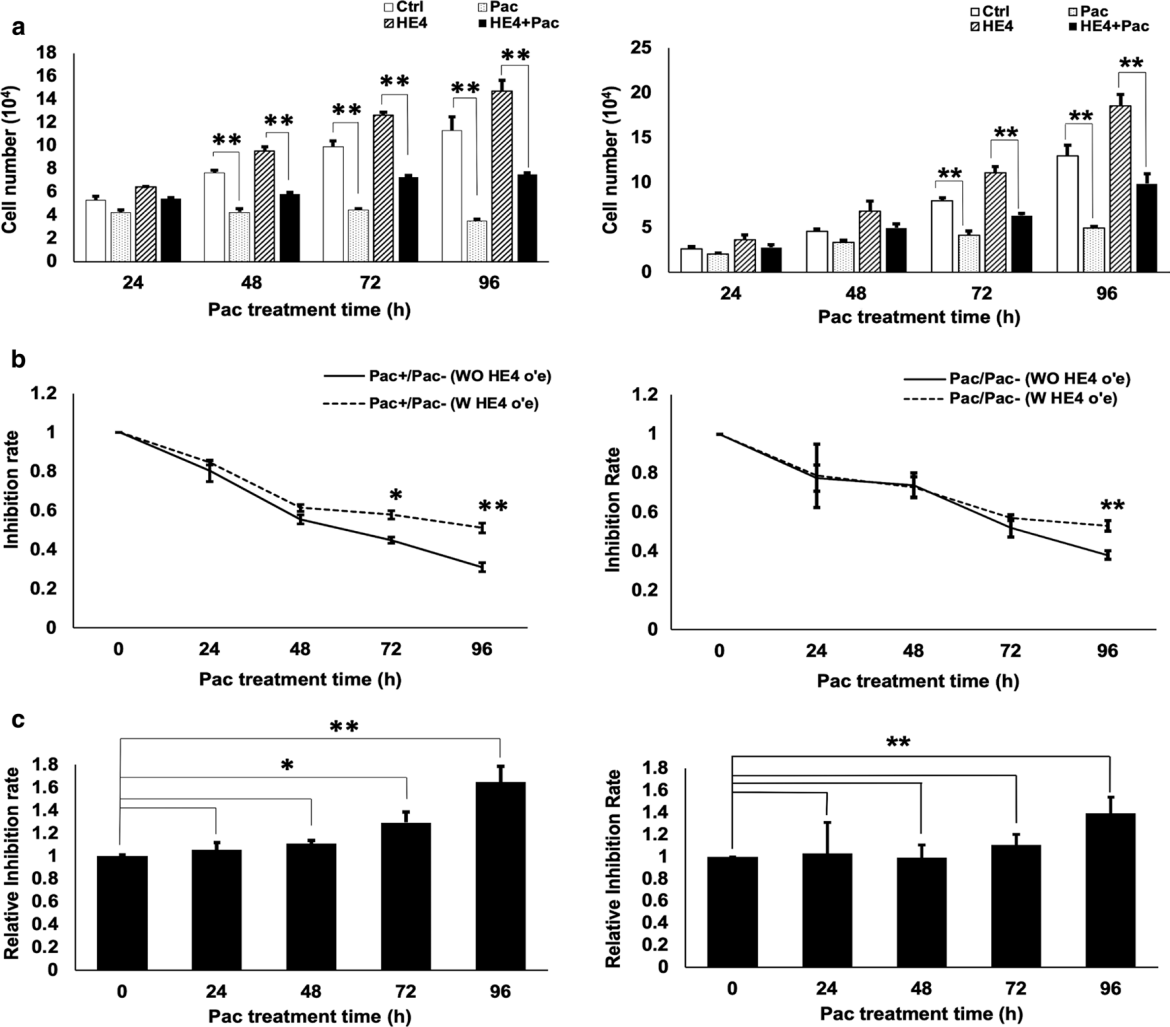
HE4 overexpression decreased the Capan-1 and Suit-2 cell sensitivity to Pac. Following cell transfection with HE4-expressing plasmid and Pac treatment, cell numbers of the four experimental groups were counted every 24 h. **a** Pac inhibited cell proliferation at each time point of post-treatment, but HE4 overexpression promoted cell proliferation. (Left Panel: Capan-1 cell line; Right panel: Suit-2 cell line). **b** The two curves showing B/A (Pac^+^/Pac^−^, without HE4 overexpression) and D/C (Pac^+^/Pac^−^, with HE4 overexpression) values became separate when Pac treatment proceeded, indicating an effect by HE4 overexpression on cell sensitivity to Pac in both cell lines (Left Panel: Capan-1 cell line; Right panel: Suit-2 cell line). **c** Value X = (D/C)/(B/A) was significantly higher than 1 compared with the control, indicating that HE4 overexpression decreased the cell sensitivity to paclitaxel, and HE4 and Pac effects were interactive (Left Panel: Capan-1 cell line; Right panel: Suit-2 cell line). *p < 0.05; ** p < 0.01

It is well recognized that Pac treatment can inhibit cell proliferation [28]. As expected, we observed a significant inhibition of Capan-1 and Suit-2 cell proliferation after treatment with 20 nM Pac for 48 h (Fig. 3a). The transient transfection of Capan-1 cells with HE4-expressing plasmid achieved a significantly increased HE4 protein level (Fig. 1). By comparing HE4 overexpression to control groups, we observed a significant promotion of cell proliferation by HE4 overexpression (Fig. 3a). This HE4 effect has been reported previously by this and other groups [27, 29, 30]. Importantly, the two curves representing B/A and D/C values at different time points separated from each other (Fig. 3b), and the X values were significantly higher than 1 (Fig. 3c), supporting an interaction between HE4 overexpression and drug effect. Thus, HE4 overexpression counteracted the drug inhibition of cell proliferation, or HE4 overexpression decreased the cell sensitivity to Pac.

**Fig. 4:**
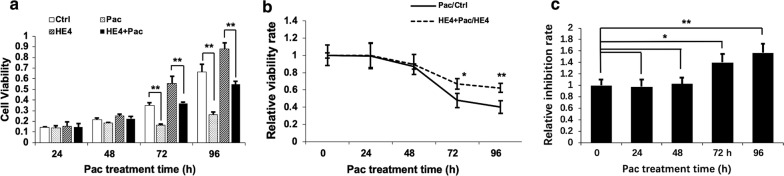
HE4 overexpression decreased Capan-1 cell sensitivity to Pac-caused inhibition of cell viability. MTT assays were performed at 24, 48, 72, and 96 h time points. **a** Results of the four experimental groups at different time points. **b** D/C and B/A values were plotted against time. Note the separation of the two curves at 72 and 96 h time points. **c** The X values increased significantly over the control after 48 h of Pac treatment. *p < 0.05; ** P < 0.01

To verify the effect of HE4 overexpression on Pac sensitivity, MTT cell viability assay was performed in Capan-1 cells. The cells were transfected with pcDNA 6.0 vector or HE4-expressing plasmid before treatment with 20 nM Pac. At 24, 48, 72, and 96 h, MTT assay was carried out to assess cell viability in the presence or absence of HE4 overexpression. As shown in Fig. 4a, Pac treatment significantly decreased cell viability compared to the control group, but HE4 overexpression significantly increased cell viability at 48, 72, and 96 h time points. Calculation and plotting of the D/C and B/A values (Fig. 4b) showed that the two curves tend to separate when the treatment time extended. Indeed, the X values were significantly higher than 1 at 72 h (p < 0.05) and 96 h (p < 0.01) (Fig. 4c), confirming that HE4 overexpression modulated the cell sensitivity to Pac.

### HE4 overexpression affected Pac inhibition of DNA synthesis

**Fig. 5:**
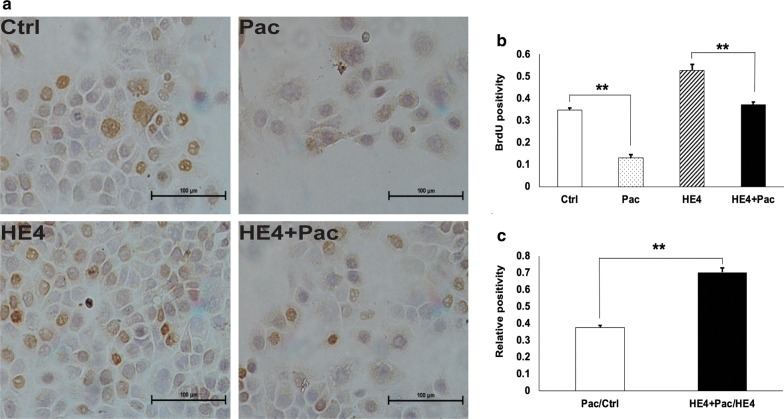
HE4 overexpression decreased the Capan-1 cell sensitivity to Pac-caused inhibition of DNA synthesis. **a** Representative results of BrdU incorporation assay in the four groups of control, HE4, Pac, and HE4 + Pac. **b** BrdU positivity was calculated for each experimental group. **c** B/A and D/C values were significantly increased with HE4 overexpression. The higher than one X value suggested that HE4 overexpression decreased cell sensitivity to paclitaxel, and the HE4 overexpression and paclitaxel effects were interactive. *p < 0.05; ** p < 0.01

BrdU was used to label S-phase cells, and BrdU positive cells were counted and positivity rates were determined (Fig. 5). Comparison of BrdU positivity among the four experimental groups indicated that Pac treatment alone inhibited, while HE4 overexpression alone promoted, DNA synthesis. The D/C (HE4 + Pac group verses HE4 group) value was significantly lower than B/A (Pac group versus control group) (X > 1), suggesting that HE4 overexpression decreased the cell sensitivity to Pac (Fig. 5c). These results confirmed that HE4 interfered with the drug-mediated inhibition of DNA synthesis, and the HE4 and drug actions were interactive.

### HE4 overexpression decreased cell sensitivity to Pac via regulating the cell cycle

**Fig. 6:**
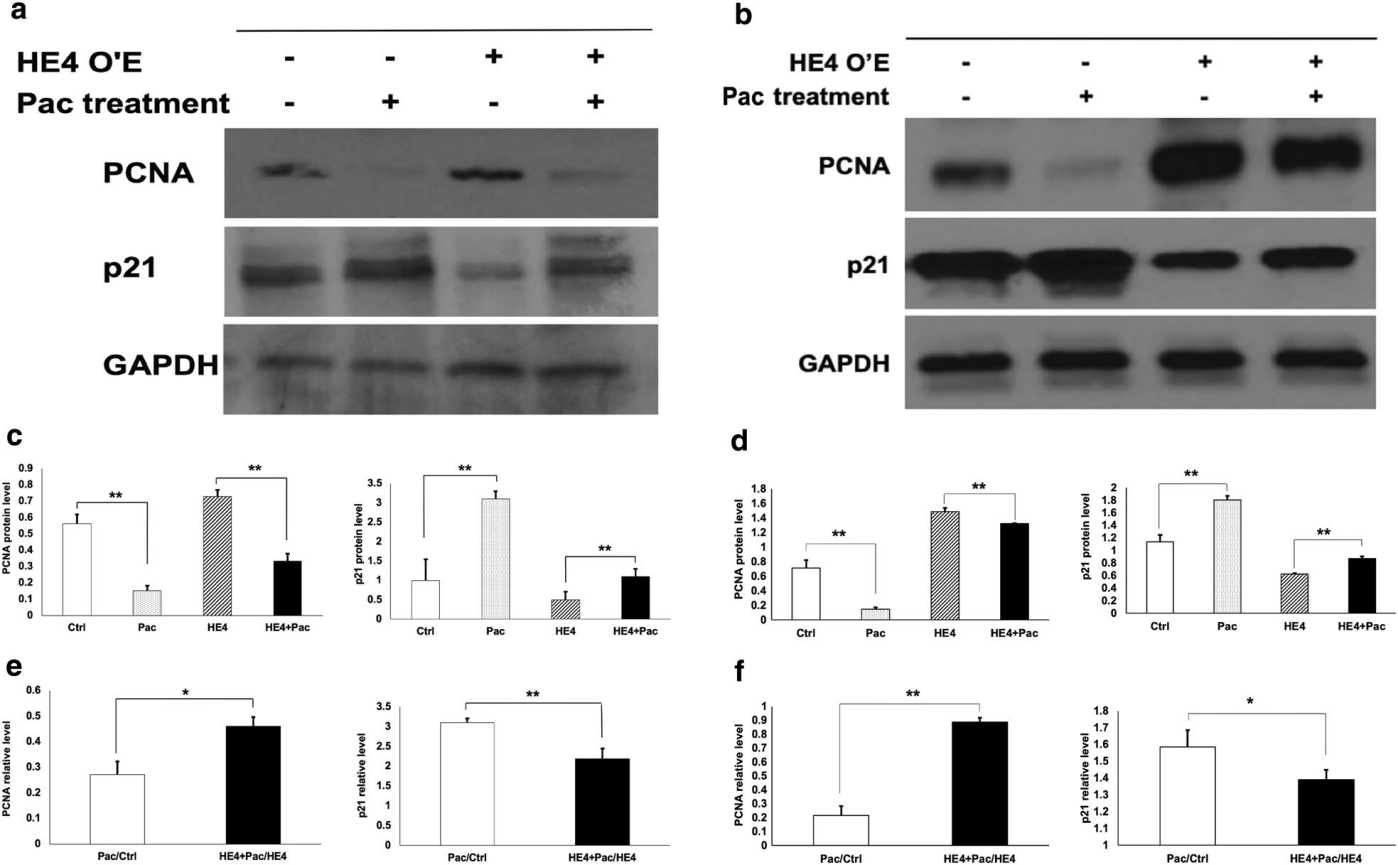
HE4 overexpression and Pac treatment affected the expression of cell cycle regulators PCNA and p21. **a** (Capan-1 cell line), and **b** (Suit-2 cell line), representative results of Western blotting with the use of specific antibodies against PCNA, p21, or GAPDH, respectively, for the four groups of control, HE4, Pac, and HE4 + Pac. **c** (Capan-1 cell line) and **d** (Suit-2 cell line), results of densitometry analysis showing the relative protein levels of PCNA (left panel) or P21 (right panel), respectively. **e** (Capan-1 cell line) and **f** (Suit-2 cell line), the significant changes of X values for PCNA (left panel) or p21 (right panel) with HE4 overexpression indicated interactive effects between HE4 overexpression and paclitaxel treatment. *p < 0.05; ** p < 0.01

To confirm the HE4 interaction with drug effects and investigate the possible mechanism, Western blotting analysis was performed to determine the protein alterations of PCNA and p21, two important cell cycle regulators, following HE4 overexpression and/or drug treatment. GAPDH protein was determined in each blot and the results provided protein loading controls (Fig. 6a). The density of each representative protein band on the blots was quantified by densitometry. Relative levels of PCNA and p21 proteins were calculated (Fig. 6b).

Following Pac treatment, the relative expression level of PCNA protein was significantly decreased. On the other hand, HE4 overexpression led to an increased expression of PCNA, indicating an HE4-mediated promotion of the cell cycle. Opposite changes to those of PCNA following Pac treatment and HE4 overexpression were observed in p21 protein, consistent with the inhibitory function of p21 in cell cycle regulation. The D/C and B/A values for both PCNA and p21 were significantly different, in other words, their quotients deviated from 1, indicating an interactive action of HE4 overexpression and Pac in the modulation of these cell cycle regulators.

### HE4 knockdown affected Pac inhibition of DNA synthesis

**Fig. 7:**
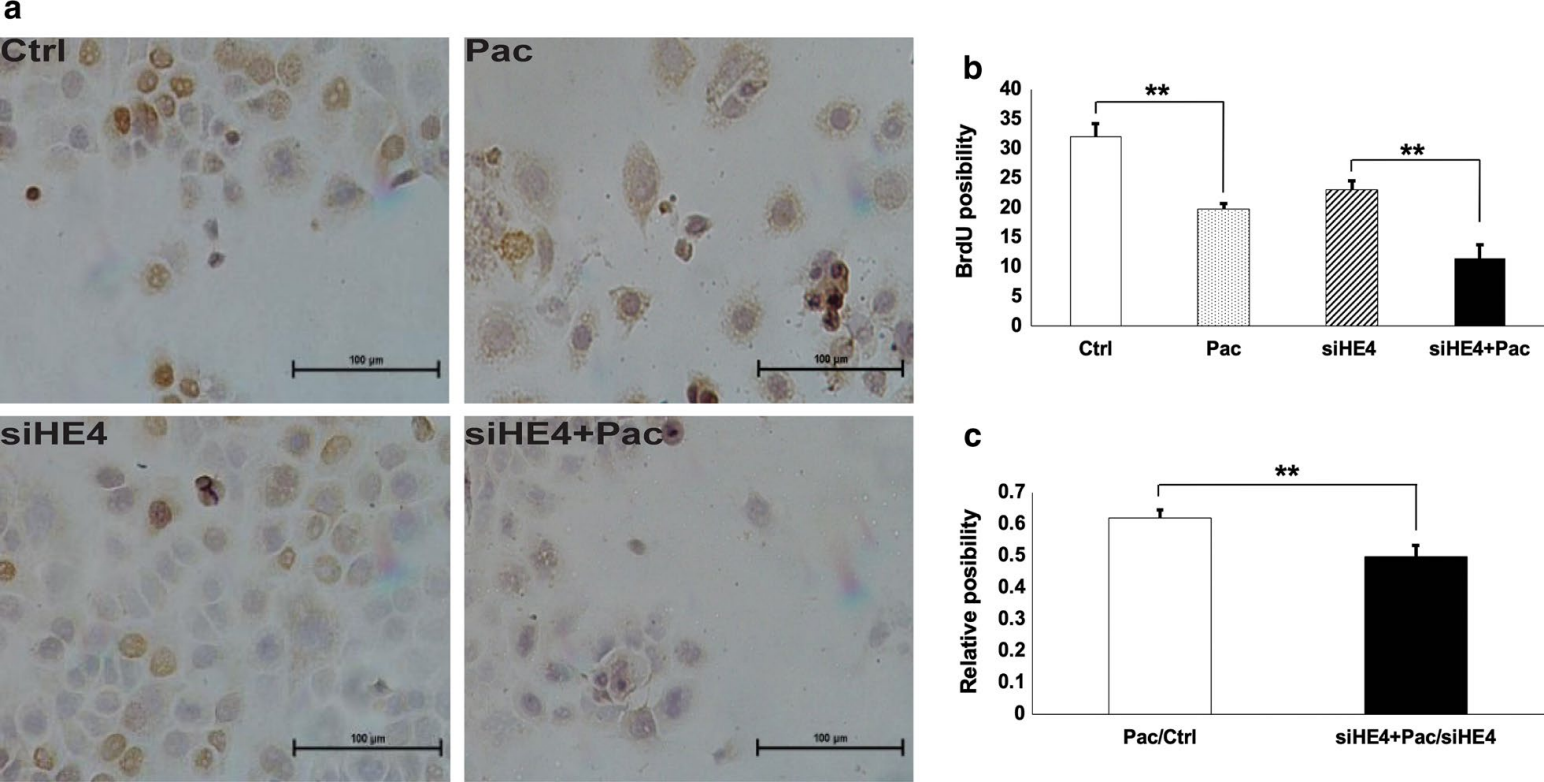
HE4 knockdown increased the Capan-1 cell sensitivity to Pac-caused inhibition of DNA synthesis. **a** Representative results of BrdU incorporation assay in the four groups of control, HE4, Pac, and HE4 + Pac. **b** BrdU positivity was calculated for each experimental group. **C.** B/A and D/C values were significantly different. The lower than one X value suggested that HE4 knockdown increased cell sensitivity to paclitaxel, and the HE4 knockdown and paclitaxel effects were interactive. *p < 0.05; ** p < 0.01

HE4 knockdown experiments were performed to confirm the role of HE4 for cell sensitivity to Pac. siRNA against HE4 was designed and transfected into Capan-1 cells, and the reduction in HE4 protein expression was verified by Western blotting (Fig. 2). In the BrdU incorporation assay, Pac treatment caused an inhibition of DNA synthesis, and HE4 knockdown led to a decreased DNA synthesis, confirming their actions on cell cycle regulation (Fig. 7). The D/C and B/A values were significantly different, and the X value was lower than 1, suggesting HE4 knockdown increased the cell sensitivity to Pac through interactive actions.

### HE4 knockdown increased cell sensitivity to Pac via regulating the cell cycle

**Fig. 8:**
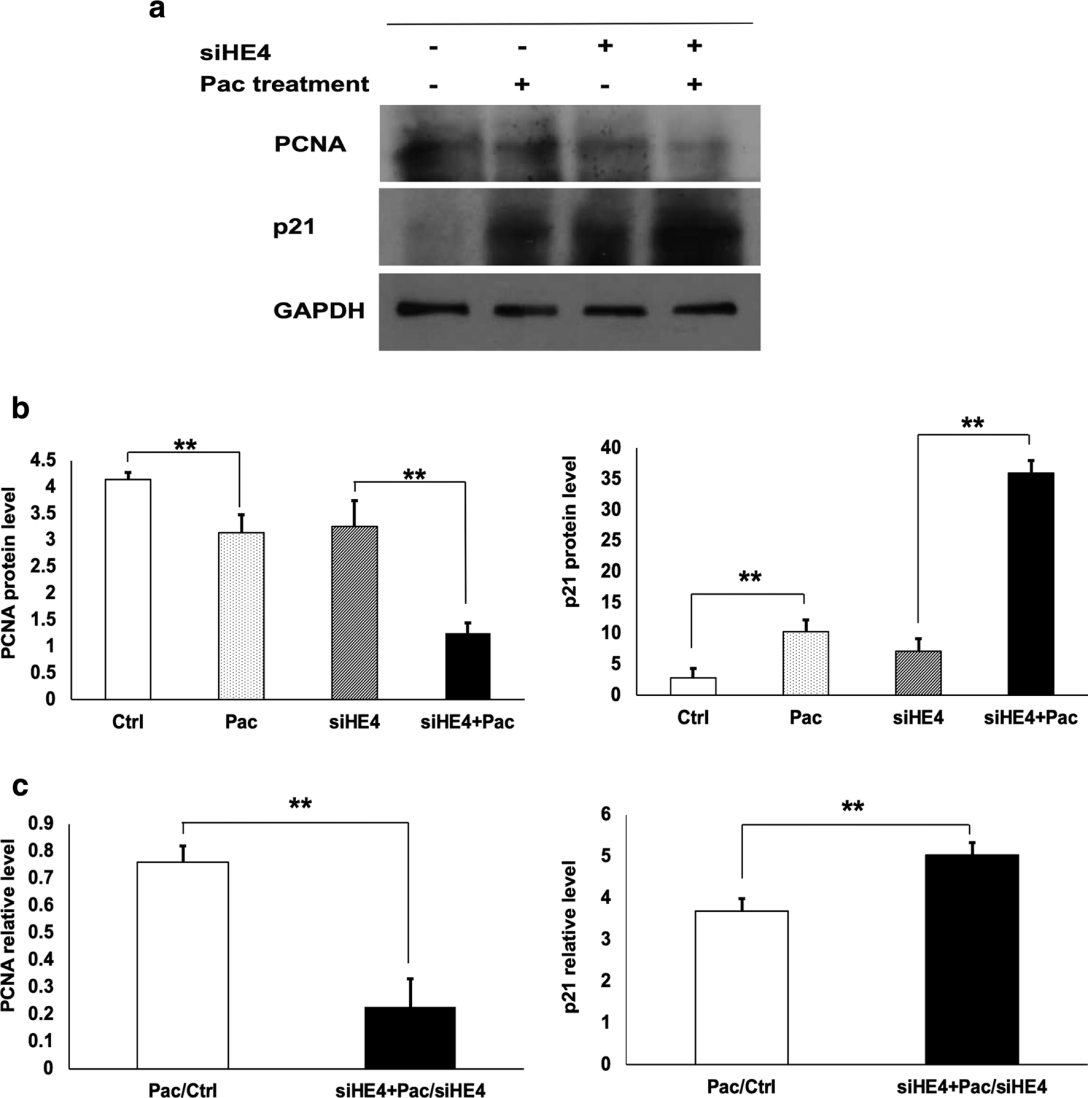
HE4 knockdown and Pac treatment affected the expression cell cycle regulators PCNA and p21 in Capan-1 cells. **a** Representative results of Western blotting with the use of specific antibodies against PCNA, p21, or GAPDH, respectively, in the control, HE4, Pac, and siHE4 + Pac experimental groups. **b** Densitometry analysis results showing the relative protein levels of PCNA (left panel) or P21 (right panel). **c** The significant changes of X values for PCNA (left panel) or p21 (right panel) with HE4 knockdown, indicating interactive effects by HE4 knockdown and paclitaxel treatment. *p < 0.05; ** p < 0.01

Western blotting was performed to determine the changes in PCNA and p21 proteins following Pac treatment and HE4 knockdown (Fig. 8). Results of densitometry analysis demonstrated decreased PCNA and increased p21 proteins by Pac treatment or HE4 knockdown, indicating that both Pac treatment and HE4 knockdown may affect cell proliferation through modulation of cell cycle regulators. Moreover, the X values for PCNA and p21 significantly deviated from 1, suggesting that a low HE4 level might act synergistically with Pac for the inhibition of the cell cycle. It should be mentioned that similar changes in p21 and PCNA were also observed in Suit-2 cells. Thus, consistent with the observations in HE4 overexpression experiments, a decreased HE4 level led to increased Capan-1 sensitivity to Pac through its effect on cell cycle regulator PCNA and p21 in both cell lines.

### HE4 overexpression in PDAC tissues

**Fig. 9:**
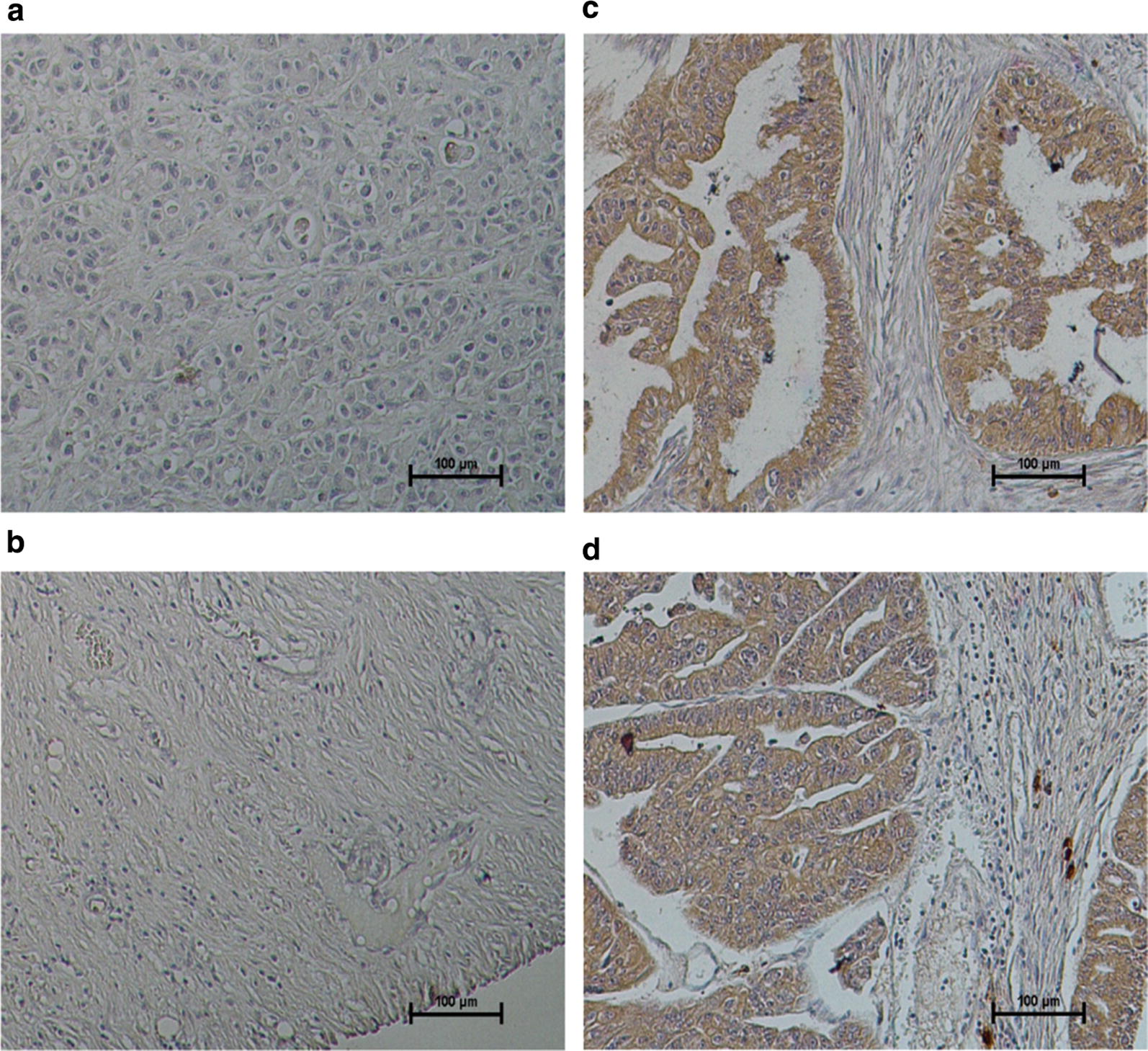
HE4 overexpression in PDAC tissues. Immunohistochemistry was performed on a PDAC microarray using the HE4-specific antibodies. **a**, **b** Two normal pancreatic tissues without negative HE4 staining. **c**, **d** High HE4 expression was detected in the two PDAC tissues. High HE4 expression was found in the glandular cancer cells, whereas low or no HE4 expression was found in the stromal compartment. HE4 protein mostly localized in the cytoplasm rather than nuclei. Representative images were taken at 20× magnification. Cell nuclei were highlighted in purple color by Hematoxylin staining. After color development with DAB, HE4 staining was shown in brown color

To verify the HE4 overexpression in pancreatic cancer cells, we performed immunohistochemistry on a PDAC tissue array containing PDAC tissues as well as normal pancreatic tissues. As shown in Fig. 9. While normal pancreatic tissues are negative for HE4 staining (Fig. 9a, b), the two PDAC tissues displayed strong positivity (Fig. 9c, d). Moreover, high HE4 expression was found in the glandular cancer cells, whereas very low or no HE4 expression could be detected in the stromal compartment. In cancer cells, HE4 protein appeared to localize predominantly in the cytoplasm rather than the nuclei. These results provided support for in vivo HE4 overexpression in pancreatic cancer cells.

## Discussion

It was observed that HE4 expression levels in malignant tissues are positively correlated with tumor sizes and stages, invasion and metastasis, and negatively correlated with patient survival, in ovarian as well as endometrial cancers [31–33]. In pancreatic cancers, higher HE4 expression levels in tumor tissues are closely correlated with larger tumor sizes and more advanced stages of malignancies [24]. Previous in vitro studies have also shown that overexpression of HE4 promoted endometrial cancer cell proliferation. Moreover, HE4 overexpression was found to enhance the growth of mouse xenografts formed by endometrial cancer cell lines [25]. Consistent with these reports, data from the current study indicate that HE4 can promote the proliferation of pancreatic cancer cells as well. All these data point to the presence of HE4 bioactivity in various malignancies. It is noteworthy that *Angioli* et al. conducted a follow-up study in 76 patients with aggressive epithelial ovarian cancer (EOC), and found that higher serumHE4 levels were associated with carboplatin resistance [34]. In the third chemotherapy cycle, EOC patients classified as platinum-resistant tended to display higher serum HE4 levels than patients classified as platinum-sensitive/intermediate. All 36 platinum-resistant cases had serum HE4 levels > 70 pM, whereas only 6 out of 40 nonresistant cases showed HE4 levels > 70 pM (Sensitivity 100%; Specificity 85%) [34]. Similarly, the prediction of chemo-response using postsurgical reduction of serum HE4 levels reached a sensitivity of 83% and a specificity of 87% (Positive predictive value = 0.86; Negative predictive value = 0.85). Thus, serum HE4 levels may be useful in predicting EOC patients’ response to chemotherapy. In this study, we observed that HE4 levels had an impact on pancreatic cancer cell sensitivity to paclitaxel. As paclitaxel, like platinum, was shown to inhibit cell cycle progression [35], our observation appears to be following the above clinical data, supporting a deep involvement of HE4 in the cell responses to chemotherapy drugs, most likely via an action in cell cycle regulation. Considering the overexpression of HE4 in many PDAC cases, the revelation of this negative HE4 effect on drug sensitivity may carry strong clinical implications for the efforts to improve the treatment of PDAC patients. HE4 could be a potential therapeutic target for the sensitization of pancreatic cancer cells to paclitaxel treatment. Additionally, HE4 expression levels may be used to predict the sensitivity of pancreatic cancer patients to paclitaxel.

**Fig. 10:**
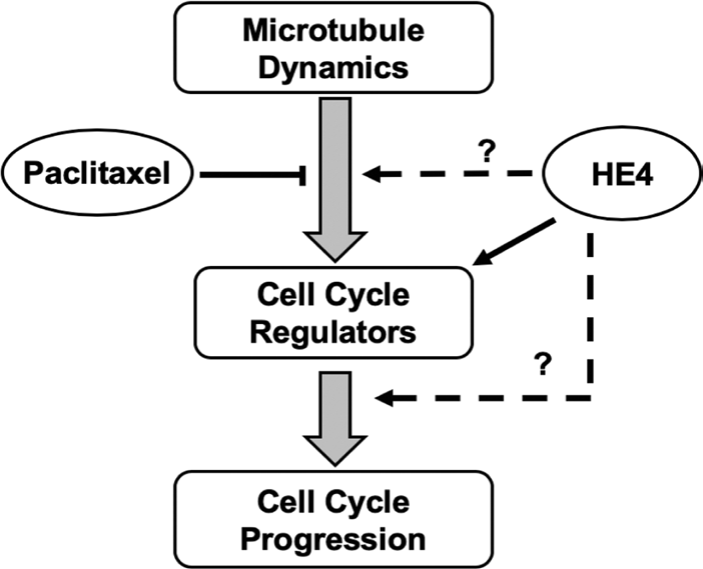
Illustration of potential mechanisms by which HE4 may affect cell sensitivity to Pac. Paclitaxel stabilizes the microtubule and protects it from disassembly, leading to cell cycle arrest and inhibition of cell proliferation as reflected by concomitant changes in cell cycle regulators of PCNA and p21. On the other side, HE4 may modulate cell cycle factors through its protease inhibitory action on paclitaxel-related cell cycle regulators. Alternatively, HE4 could affect other factors up- and down-stream of PCNA and p21. In any case, HE4 function may impact the cell sensitivity to paclitaxel through an action(s) related to paclitaxel, and these effects would converge to a pathway most possibly governing the cell cycle progression

Analysis of HE4 protein structure suggested that HE4 contains a highly conserved WAP (Whey Acidic Proteins) domain composed of four disulfide core regions harboring eight cysteines [16, 19]. It has been proposed that the inhibitory loop of the WAP domain can be inserted into the active site of a variety of proteases to inhibit their catalytic activities. LeBleu et al. [36] performed a series of biochemical experiments to demonstrate a strong serine protease inhibitor activity of purified HE4 protein. Overexpression of Elafin, another member of the WAP domain family exhibiting a protease inhibitor activity, was found to increase the cisplatin resistance in the ovarian cancer cell line SKOV3 [37]. The common involvement of WAP domain factors in drug sensitivity points to the possibility that these effects could be mediated by the protease inhibitor activity. In this study, we observed that while HE4 overexpression could counteract the paclitaxel-caused inhibition of cell proliferation, HE4 knockdown enhanced the drug effects. Moreover, HE4 appeared to affect drug sensitivity through modulation of cell cycle regulators such as PCNA and p21 in both Capan-1 and Suit-2 PDAC cell lines. As illustrated in Fig. 10, it is possible that HE4 directly inhibited a protease responsible for the degradation of PCNA and/or other cell cycle regulators to promote DNA synthesis. Alternatively, the HE4 protease inhibitor activity could affect other factors up- or down-stream of DNA synthesis. For example, Paclitaxel was known to impede the cell cycle progression by stabilizing the microtubule. HE4 could affect the function of a factor(s) involved in microtubule assembly to directly counteract the effect of Pac. In any case, the HE4 effects would converge with those of paclitaxel on the level of cell cycle regulation. Further studies are required to determine the exact mechanism of HE4-mediated changes in paclitaxel sensitivity.

The four-group experimental design and calculation of the “X” value represent the strength of this study, which enables us to quantitatively evaluate the interaction between HE4 and paclitaxel actions. However, the current study has several limitations. Firstly, although we demonstrated the overexpression of HE4 in the cancer cells by immunohistochemistry studies on PDAC, the use of only two cell lines and the lack of in vivo studies using in animal model limits the extrapolation of the observations. Secondly, Paclitaxel-treated cells exhibited defects in mitotic spindle assembly and chromosomes segregation [38]. Blocking of mitosis and prolonged activation of the mitotic checkpoint could trigger apoptosis [39]. How HE4 and Pac by themselves or in combination may affect cell apoptosis was not addressed in the current study. Thirdly, previous studies have shown an important role of P-gp-mediated multi-drug resistance mechanism for cell response to paclitaxel [40, 41]. Although in preliminary experiments we explored the expression changes of several multi-drug resistance genes and did not detect significant alterations, their involvement could not be excluded. Lastly, drug concentrations were optimized to observe a maximal HE4 effect on cell responses to paclitaxel. It remains unclear how much these conditions could recapitulate the in vivo situation, e.g. the HE4 overexpression levels as well as the local drug concentrations in cancer tissues of PDAC patients. Moreover, the Ras/MAPK signaling cascade is well recognized for its significance in the regulation of pancreatic cancer behaviors. How this important pathway may be involved in the observed HE4 and Pac interaction remains unclear. Further in vivo and mechanistic studies, including animal model experiments and clinical investigations on HE4 correlation to drug sensitivity/resistance, are required to better understand the interaction of HE4 function and paclitaxel effects on a molecular level.

## Conclusion

In summary, HE4 overexpression in PDAC tissues suggested that HE4 could be a biomarker for the detection of PDAC. Capan-1 and Suit-2 pancreatic cancer cell cultures were used to investigate the HE4 involvement in PDAC cell response to paclitaxel. Results of cell counting,MTT cell proliferation and BrdU incorporation assays consistently supported that HE4 did not only affect cell proliferation, but also affected cell sensitivity to paclitaxel treatment. These effects appeared to be mediated by the HE4 regulation of PCNA and p21, most likely through its protease inhibitor action. These findings suggested that HE4 could be a potential therapeutic target for delivering more effective paclitaxel therapy to pancreatic cancer patients. The

## Data Availability

The data for this study are all included in the article.

## Abbreviations

PDAC: Pancreatic ductal adenocarcinoma
HE4: Human epididymis protein 4
Pac: Paclitaxel
WAP: Whey-acidic-protein
WFDC: WAP-type four-disulfide core
